# Medicinal cannabis for treating post-traumatic stress disorder and comorbid depression: real-world evidence

**DOI:** 10.1192/bjo.2024.13

**Published:** 2024-03-12

**Authors:** Michael T. Lynskey, Alkyoni Athanasiou-Fragkouli, Hannah Thurgur, Anne Katrin Schlag, David J. Nutt

**Affiliations:** Drug Science, London, UK; Psychedelic Research Group, Centre for Neuropsychopharmacology, Division of Brain Sciences, Faculty of Medicine, Imperial College London, UK; and Drug Science, London, UK

**Keywords:** Post-traumatic stress disorder, comorbidity, depressive disorders, medicinal cannabis, treatment outcome

## Abstract

**Background:**

Cannabis-based medicinal products (CBMPs) are increasingly being used to treat post-traumatic stress disorder (PTSD), despite limited evidence of their efficacy. PTSD is often comorbid with major depression, and little is known about whether comorbid depression alters the effectiveness of CBMPs.

**Aims:**

To document the prevalence of depression among individuals seeking CBMPs to treat PTSD and to examine whether the effectiveness of CBMPs varies by depression status.

**Method:**

Data were available for 238 people with PTSD seeking CBMP treatment (5.9% of the treatment-seeking sample) and 3-month follow-up data were available for 116 of these. Self-reported PTSD symptoms were assessed at treatment entry and at 3-month follow-up using the PTSD Checklist – Civilian Version (PCL-C). The probable presence of comorbid depression at treatment entry was assessed using the nine-item Patient Health Questionnaire (PHQ-9). Additional data included sociodemographic characteristics and self-reported quality of life.

**Results:**

In total, 77% met screening criteria for depression, which was associated with higher levels of PTSD symptomatology (mean 67.8 *v.* 48.4, *F*_(1,236)_ = 118.5, *P* < 0.001) and poorer general health, quality of life and sleep. PTSD symptomatology reduced substantially 3 months after commencing treatment (mean 58.0 *v.* 47.0, *F*_(1,112)_ = 14.5, *P* < 0.001), with a significant interaction (*F*_(1,112)_ = 6.2, *P* < 0.05) indicating greater improvement in those with depression (mean difference 15.3) than in those without (mean difference 7).

**Conclusions:**

Depression is common among individuals seeking CBMPs to treat PTSD and is associated with greater symptom severity and poorer quality of life. Effectiveness of CBMPs for treating PTSD does not appear to be impaired in people with comorbid depression.

Cannabis-based medicinal products (CBMPs) are increasingly available internationally and are utilised for a range of chronic conditions, including chronic pain and anxiety disorders.^1,2^ One diagnosis which is among the more common reasons for seeking medicinal cannabis is post-traumatic stress disorder (PTSD).^1,3,6^ For example, Sznitman^6^ reported a steady increase in the percentage of medical cannabis licenses that were issued to treat PTSD in Israel: by 2018, this represented the third highest target indication for receiving a medical cannabis license, accounting for 10.3% of all licenses granted. In a large US-based sample (>60 000) of medicinal cannabis users, Mahabir et al^5^ reported that PTSD was the third most common primary medical condition (8.4% of all patients). Similarly, we previously reported that PTSD was the third most common condition (6.1% of patients) reported by UK medical cannabis patients in our Project Twenty21 initiative^1^ (see below).

## Medical cannabis: ingredients and mechanism of action

There are numerous lines of evidence supporting the potential of CBMPs for treating PTSD, including preclinical evidence suggesting mechanisms that may explain benefits of prescribed cannabis.^7,8^ The main functional ingredients of medical cannabis are delta-9-tetrahydrocannabinol (THC) and cannabidiol (CBD). The former is responsible for the psychoactive effects (colloquially referred to as ‘stoned’) of recreational cannabis, but THC also has powerful analgesic effects^9^ that are likely mediated via stimulation of cannabinoid 1 (CB1) receptors on glutamate neurons. Trauma-related disorders such as PTSD can be considered as disorders of the endogenous (endocannabinoid) systems that also work through CB1 receptors. Such a deficit might be to some extent rectified by exogenous THC, which in addition also improves sleep^10^ so can reduce the disruption and distress of nightmares. CBD is itself anxiolytic^11^ and has powerful anti-epilepsy effects which presumably relate to reducing central glutamate excitability, something that is likely to be enhanced in cases of PTSD with flashbacks and nightmares. Although it is not yet understood what the mediating brain mechanisms of CBD are, they may involve increased production of endocannabinoids that have homeostatic or adaptogenic function.^12^

## Evidence base for use in PTSD

Given the increasing adoption of CBMPs as a treatment for PTSD there have been surprisingly few clinical trials on the efficacy of CBMPs for this indication. Several comprehensive and influential systematic reviews^13^ have been able to identify only one randomised controlled trial (RCT) of CBMPs for PTSD and, although this showed promising effects of nabilone in improving global functioning and reducing nightmare frequency, it was based on only 10 (male) participants and did not include any assessment of PTSD symptomatology.^14^ Since then, one further RCT of smoked cannabis which employed a cross-over design reported that although different cannabis preparations were well tolerated and were associated with improvements in symptomatology, the level of improvement was no greater than that experienced when using placebo.^15^ Thus, there is little, if any evidence from clinical trials to support the use of CBMPs to treat PTSD.

Nonetheless, real-world studies capitalising on the widespread use of medicinal cannabis to treat PTSD have reported promising results: in our own Project Twenty21 registry study^1^ we reported that, after 3 months, there had been a substantial reduction in symptoms of PTSD (estimated effect size, Cohen's *d* = 0.69; 95% CI 0.42–0.95) assessed using the PTSD Checklist – Civilian Version (PCL-C).^16^ Similarly, Pillai et al^17^ reported reductions in PTSD symptomatology after commencing CBMP treatment, with effects sizes at 1, 3 and 6 months varying between 0.30 and 0.50 for the avoidance, intrusion and hyperarousal subscales of the Impact of Event Scale – Revised (IES-R), as well as for the total IES-R score.^18^ Nacasch et al^19^ also reported positive effects of CBMPs for people with PTSD, albeit in a smaller sample.

Given promising preliminary findings about the potential utility of CBMPs for PTSD and their growing use for this indication despite a relative lack of high-quality empirical research testing their efficacy and effectiveness, there is a need for more research, including both trials and real-world studies, of the effects of these drugs. Although RCTs are typically considered the gold standard of medical evidence^20^ they are not without shortcomings:^21^ they can be prohibitively expensive and are typically based on relatively small sample sizes with brief periods of treatment. Further, they typically apply extensive exclusion criteria, an approach that may threaten the external validity of findings from these trials.^20^ Specifically, it is common practice for trials focusing on a specific condition to exclude individuals with comorbid psychiatric or other health conditions.^22,23^

However, epidemiological research has consistently shown that there are high levels of comorbidity between PTSD and major depressive disorder (among other disorders), with estimates typically suggesting that a minimum of half those with PTSD will also meet criteria for major depressive disorder, and these rates are likely to be even higher in treatment-seeking samples.^24^ If future trials exclude patients with major depressive disorder they may inadvertently study a sample of people unrepresentative of those seeking treatment, with the consequence that their results may not be representative of treatment effects that would be obtained in the real world.

## The current study

Given this background we documented the prevalence of depressed mood in a sample of people seeking CBMPs for the treatment of PTSD, compared characteristics of those with and without comorbid depressed mood and examined whether 3-month outcomes varied between the two groups. These issues are examined using data from Project Twenty21 (T21), a large ongoing registry of people seeking CBMPs privately in the UK.

## Method

### Project Twenty21

Launched in August 2020, Project Twenty21 (T21) seeks to develop real-world evidence on the effectiveness and safety of medical cannabis.^1,2,25^ T21 is a multi-centre, prospective, observational patient registry of real-world data that includes data from patients in the UK receiving medical cannabis for a variety of conditions. Patients entered into the registry are followed up at 3-monthly intervals.

### Recruitment strategy and consent

An individuals must have an established diagnosis and evidence of failure of at least two treatment approaches before being eligible to legally receive prescribed CBMPs in the UK. Only specialist medical practitioners can prescribe CBMPs but patients can self-refer to a prescribing physician.

Patients making an appointment at one of the clinics affiliated with T21 were invited to participate in the registry. There were no inclusion or exclusion criteria specifically for participation in the registry: decisions about the suitability of CBMPs for a specific individual were entirely the responsibility of the treating physician. All individuals agreeing to participate in the registry provided written consent.

Prescribers partnering with T21 have access to a formulary that contains a range of CBMPs, including oils and flower of differing CBD and THC concentrations. However, there are no restrictions on what products can be prescribed and products from outside the formulary are also prescribed.

Between the start of the project (August 2020) and 1 November 2023 a total of 4036 individuals had contributed data to T21. During the first phase of the project, patients were recruited only if they were seeking treatment for any of eight categories of primary condition: anxiety disorders, chronic pain, multiple sclerosis, PTSD, Tourette syndrome, epilepsy, substance use disorder and attention-deficit hyperactivity disorder. However, from February 2022 all patients receiving a prescription of CBMPs were eligible to participate in the registry, regardless of their primary condition. The most common primary conditions were chronic pain (49.4% of the sample), anxiety disorders (42.4%) and PTSD (5.9%).

### Measures

As part of their clinical assessment, in addition to providing information on their medical history, including past and current treatments, patients complete structured assessments of symptomatology. The measures used in the current analyses are as follows.

#### Post-traumatic stress disorder

PTSD was assessed using the PCL-C,^16^ which has 17 items assessing PTSD symptomatology. Each of the 17 items is rated on a 5-point Likert scale (‘not at all’, ‘a little bit’, ‘moderately’, ‘quite a bit’, ‘extremely’) and can be summed to form a single measure of PTSD symptomatology. A preliminary test of the psychometric properties of the scale indicated it had high reliability (Cronbach's alpha 0.93) and item total correlations were all substantial, ranging from 0.38 to 0.75.^1^

#### Comorbid major depression

Mood/depression was assessed using the PHQ-9,^26^ which is widely used to assess symptoms of depression. Patients are asked to rate the occurrence of nine events in the past 2 weeks, with each question rated on a 4-point Likert scale (‘not at all’, ‘several days’, ‘more than half the days’, ‘nearly every day’). The scale produced by summing responses to these items, which can range from 0 to 27, has been shown to perform adequately as a screener for major depressive disorder, with scores of 10 or higher suggestive of a probable diagnosis of major depression.

### Patient characteristics

#### Gender

Self-reported gender was coded as male, female or non-binary.

#### Age

Age at the time of initiation of treatment, expressed in years, was included as a continuous variable.

#### Self-reported health

All participants were asked to complete a series of self-report measures assessing aspects of health and quality of life. These measures were based on widely used and well-validated assessments. A full description of the measures used and their psychometric properties in the current sample has been provided previously.^1^ In the current report we analysed general measures of health assessed across all primary conditions:

- quality of life, assessed using the five items of the EQ-5D-5L^27^
- general health, assessed using the visual analogue scale (VAS) of the EQ-5D-5L^27^
- sleep quality, assessed using four items derived from the Pittsburgh Sleep Quality Index (PSQI).^28^

### Prescribed CBMPs

The medical record contained information on what CBMPs were prescribed for each individual. We report information on the number of products prescribed for each participant. Further, we report the total number of different products prescribed to all participants and classify these according to their form (oil or flower) and relative THC and CBD ratios (CBD dominant, balanced and THC dominant). The percentage of prescriptions falling into each of these six categories is reported.

### Statistical analysis

First, we examined the prevalence of our probable diagnosis of major depressive disorder in the sample of people with a primary diagnosis of PTSD and compared severity of PTSD symptomatology, sociodemographic characteristics (age, gender) and measures of general well-being between the two groups. Differences in categorical values (e.g. gender) were assessed using the chi-squared (χ^2^) statistic, and differences in mean ratings (e.g. quality of life) were conducted using one-way analysis of variance (ANOVA). These analyses were conducted on all participants with PTSD as their primary condition who have data available at baseline.

We compared the total number of CBMPs prescribed to the two groups using one-way ANOVA and the percentage of participants in each group receiving the six categories of CBMP described above (CBD-dominant oil, balanced oil, THC-dominant oil, CBD-dominant flower, balanced flower, THC-dominant flower) using χ^2^.

We then compared changes in the severity of PTSD symptomatology between starting treatment and 3-month follow-up for those with or without secondary depression. Repeated-measures general linear models were used to estimate the between-individuals factor of depression, the within-individual factor describing changes between baseline and follow-up and the interaction between these two factors. These analyses include age and gender as covariates.

All analyses were conducted in SPSS version 29.0.1 for Windows.^29^

### Ethics

According to the National Health Service Health Research Authority, Project T21 is classified as research; however, based on the Medical Research Council decision tools, Research Ethics Committee review and approval is not required. All individuals did, however, provide signed informed consent for their data to be used for research purposes.

## Results

### The prevalence of PTSD and comorbidity with depression

As of 1 May 2023, there were 4036 people who had contributed data to T21: 238 (5.9%) of these reported that PTSD was the primary condition for which they were seeking treatment. The estimated prevalence of depression was high: 77.7% of those with PTSD met screening criteria for probable major depression.

### Comparisons between those with PTSD with and without depression

Table 1 compares the severity of PTSD symptomatology, measures of quality of life and sociodemographic characteristics between participants with PTSD with and without depression.

**Table 1:** Mean post-traumatic stress disorder (PTSD) symptom severity, quality of life and sociodemographic characteristics of PTSD patients with and without comorbid depression

	Comorbid depression^a^		*F*_(d.f.)_
	No	Yes	
**PTSD severity**			
Mean PCL-C score	48.4 (53)	67.8 (185)	*F*_(1,236)_ = 118.5***
**Quality of life**			
Quality of life (EQ-5D-5L)	0.67 (53)	0.48 (187)	*F*_(1,238)_ = 22.0***
General Health	60.9 (53)	47.1 (187)	*F*_(1,238)_ = 20.4***
Sleep	10.2 (53)	14.9 (187)	*F*_(1,238)_ = 82.3***
**Sociodemographic characteristics**			
Average age, years	39.7 (53)	39.2 (187)	*F*_(1,238)_ = 0.1^n.s.^
Gender^b^			χ^2^_1_ = 7.7**
Male	28.5% (41)	71.7% (103)	
Female	13.0% (12)	87.0% (80)	

**P < 0.01, ***P < 0.001, n.s., non-significant.

a. Numbers in parentheses show number of patients.

b. Owing to the small number identifying as non-binary (<5) they were excluded from these analyses to protect anonymity.

Participants with PTSD and comorbid depression reported substantially higher levels of PTSD symptomatology than those without depression (mean 67.8 *v.* 48.4; *F*_(1,236)_ = 118.5, *P* < 0.001). They also reported lower well-being across three indicators: quality of life (*F*_(1,238)_ = 22.0, *P* < 0.001), general health (*F*_(1,238)_ = 20.4, *P* < 0.001) and sleep (*F*_(1,238)_ = 82.3, *P* < 0.001). The prevalence of depression was higher among females (87.1%) than among males (71.7%, χ^2^_1_ = 7.7, *P* < 0.01) but there were no age differences between the two groups.

### Types of CBMPs by depression status

There were no differences between individuals with comorbid depression and remaining sample members in terms of the total number of CBMPs they were prescribed: those with depression received an average of 2.1 products whereas those without depression received an average of 2.0 products (*F*_(1,105)_ = 0.01; *P* > 0.70). The most commonly prescribed category of CBMP was THC-dominant flower, with 74.8% of our PTSD sample receiving a prescription for at least one of these products. However, there were no differences between those with or without comorbid depression in the percentages of people receiving specific types of product (all *P* > 0.10).

### Differences between those retained and those lost to follow-up

Data on PTSD symptomatology were available at treatment entry and at 3 months for 114 individuals, representing 48.3% of those for whom baseline data were available. Missing data at 3 months occur as a function of two processes: withdrawal from the study (either because participants cease medicinal cannabis use or continue using medicinal cannabis but do not contribute data to the project) and analyses occurring before all enrolled individuals have been in the study for 3 months and have therefore not yet been eligible for follow-up. Those who contributed data at 3 months did not differ from those not contributing data on any measure of symptom severity, sociodemographic characteristics or quality of life and well-being at entry, as summarised in Table 2.

**Table 2:** Mean post-traumatic stress disorder (PTSD) symptom severity, quality of life and sociodemographic characteristics of PTSD patients followed-up at 3 months and those lost to follow-up

	Followed up at 3 months^a^		*F*_(df)_
	No	Yes	
**PTSD severity**			
Mean PCL-C score	64.1 (122)	62.7 (116)	*F*_(1,236)_ = 0.6^n.s.^
**Quality of life**			
Quality of life (EQ-5D-5L)	0.51 (122)	0.53 (118)	*F*_(1,238)_ = 0.5^n.s.^
General health	50.7 (122)	49.7 (118)	*F*_(1,238)_ = 0.1^n.s.^
Sleep	13.9 (122)	13.8 (118)	*F*_(1,238)_ = 0.1^n.s.^
**Sociodemographic characteristics**			
Average age, years	39.6 (132)	39.3 (121)	*F*_(1,238)_ = 0.1^n.s.^
Gender^b^			χ^2^_1_ = 1.4^n.s.^
Male	48.6% (70)	51.4% (74)	
Female	56.5% (52)	43.5% (40)	

n.s., non-significant.

a. Numbers in parentheses show number of patients.

b. Owing to the small number identifying as non-binary (<5) they were excluded from these analyses to protect anonymity.

### Does the effectiveness of medicinal cannabis vary by depression status?

**Fig. 1:**
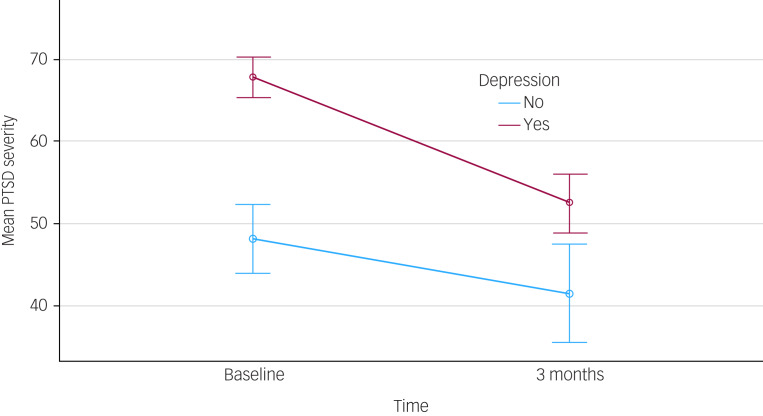
Estimated marginal means of post-traumatic stress disorder (PTSD) severity by depression status.

To test whether treatment with CBMPs reduced PTSD symptomatology at 3 months and whether any reductions varied between those with and without depression, we fit a repeated-measures generalised linear model, the results of which are summarised in Fig. 1. These analyses indicated that the severity of PTSD symptomatology was higher among those with comorbid depression (mean 60.2) than among those without depression (mean 44.8) (*F*_(1, 112)_ = 36.8, *P* < 0.001). There was a substantial within-participant reduction in PTSD severity across time (baseline mean 58.0 *v.* 3-month mean 47.0, *F*_(1,112)_ = 14.5, *P* < 0.001). Finally, there was a significant interaction between depression status and wave of data collection (*F*_(1,112)_ = 6.2, *P* < 0.05). As shown in Fig. 1, the overall reduction in PTSD symptomatology was higher in those with depression (baseline mean 67.8 *v.* 3-month mean 52.5) than in those without depression (baseline mean 48.2 *v.* 3-month mean 41.5).

## Discussion

In this sample of individuals seeking treatment with prescribed medicinal cannabis for PTSD, over three-quarters (77%) met screening criteria for probable depression. Meeting depression criteria was associated with more severe PTSD symptomatology and poorer quality of life (worse general health and quality of life assessed using the EQ-5D-5L and poorer sleep quality). Treatment with prescribed cannabis was associated with substantial reductions in the severity of PTSD symptomatology, with these improvements being more marked in individuals with depression.

### Interpretation of our findings and comparison with the literature

In this sample, treatment with CBMPs is associated with significant improvements in well-being and quality of life in PTSD after 3 months of treatment^1^ and these improvements in well-being are maintained in patients with psychiatric disorders, including PTSD, or chronic pain for up to 12 months (unpublished results: further details available from M.T.L. on request). These results parallel previous findings for quality of life across a broad range of conditions^30^ and for PTSD specifically^17^ and highlight the potential benefits of CBMPs for symptom reduction and improvements in well-being across a wide range of chronic conditions.

Importantly, our findings add to this emerging literature by indicating that the presence of comorbid depression is associated with a greater reduction in PTSD symptomatology. This finding is in sharp contrast to findings from a recent meta-analysis of trauma-focused psychotherapy for the treatment of PTSD which indicated that comorbid depression is associated with a reduced efficacy of this treatment.^31^ Although both anti-depressants, particularly SSRIs, and psychological therapies, including prolonged exposure and cognitive–behavioural therapy, have been shown to be effective for treating PTSD, many people do not respond to these treatments and there is a considerable unmet treatment need. The current results suggest CBMPs may have a role in addressing this unmet need, while there is parallel evidence indicating that psychedelics, including 3,4-methylenedioxymethamphetamine (MDMA), may also be effective in treating PTSD. Notably, Mitchell et al,^32^ who demonstrated high efficacy for MDMA, did not exclude people with major depression from their clinical trial and, in fact, reported a high prevalence (91.1%) in their sample.

The apparent increase in effectiveness of CBMPs for those with comorbid depression may reflect a number of processes. First, there may be a reciprocal or asymmetrical relationship between changes in depression symptoms and PTSD symptoms. Evidence suggests that the relationship between changes in depression and PTSD symptoms may be affected differently depending on treatment modalities.^33,34^ Further, there is preliminary evidence suggesting that CBMPs may be effective in treating depression^35^ and that reduced depressive symptomatology may be associated with a similar reduction in PTSD symptomatology. Similarly, there is some level of symptom overlap between PTSD and major depression^36^ and it may be that those transdiagnostic symptoms are especially responsive to treatment with CBMPs. Indeed, the high degree of comorbidity and symptom overlap between PTSD and major depression has led to suggestions that the comorbid condition may be a distinct subtype of PTSD.^36^ Alternatively, the greater improvement observed in those with comorbid depression may be a reflection of the greater severity of PTSD symptomatology at the start of treatment in this comorbid group.

### Implications for future trials

Our findings have implications for the design of future trials of CBMPs in the treatment of PTSD and other conditions. Specifically, it is common (although not universal) for such trials to exclude people with comorbid major depression or other psychiatric or general health conditions.^21^ The current findings suggest that this approach risks potentially recruiting a sample who are unrepresentative of patients with a specific condition (e.g. less than a quarter of patients in this sample would be eligible for participation in a trial even if the only exclusion criteria was depression). Additionally, our finding that CBMPs were significantly more effective for those with depression suggests that the application of exclusion criteria may result in the recruitment of participants who are least likely to benefit from CBMPs. This possibility may help explain the apparent discrepancy between real-world studies and RCTs, in which results from observational studies have typically reported more promising effects of CBMPS.^37^ For example, the meta-analysis reported by Black et al^13^ included trials of cannabinoids for the treatment not only of PTSD but also of a range of additional psychiatric disorders, including depression, anxiety, tic/Tourette syndrome, attention-deficit hyperactivity disorder and psychosis. They concluded that there was no evidence that pharmaceutical THC (with or without CBD) improved any psychiatric outcomes other than anxiety occurring within the context of other medical conditions, and one study suggested that it increased negative symptoms in psychosis. They also reported that pharmaceutical THC increased adverse events and withdrawals from treatment. However, medicinal cannabis prescriptions in the UK now are increasingly for whole-plant extracts that have other constituents that may also be functional via the entourage effect.^38^

### Risk of drug–drug interactions

There are concerns regarding the potential ability of CBMPs to engage in clinically significant drug–drug interactions (DDIs) with commonly prescribed medications, including antidepressants, owing to their involvement with the cytochrome P450 (CYP450) system.^39^ This is of relevance in the context of PTSD and depression as patients often concurrently use other medications. Some studies suggest that CBD and THC may inhibit CYP450 isoenzymes,^39,41^ which play crucial roles in the metabolism of many pharmaceutical drugs. This inhibition could lead to altered drug concentrations and potentially affect the efficacy and safety of concurrently used medications. Although the drug–drug interactions between cannabinoids and various drugs at the CYP level are reported *in vitro*, their clinical relevance remains unclear.^41^ Prescribers should carefully monitor patients using CBMPs in conjunction with drugs metabolised by the CYP450 pathway to mitigate the risk of adverse interactions. Real-world evidence can play a key role in understanding the clinical relevance of these interactions and contribute to the development of informed clinical guidelines for the concurrent use of CBMPs and other medications for depression/PTSD.

### Limitations

Results from our analyses should be interpreted in light of several potential limitations, including lack of a placebo control. Owing to the relatively small sample size, we were also unable to consider the broader range of chronic conditions that may be comorbid with PTSD and the extent to which the effectiveness of CBMPs may vary with multimorbidity and other specific comorbidities. Further, there was substantial attrition, consistent with other observational studies like this one. Nonetheless, our analyses indicated that the included sample did not differ from those lost to follow-up in terms of severity of PTSD symptomatology, well-being or sociodemographic characteristics.

There appears to be a surprising disconnect between the widespread use of medicinal cannabis to treat PTSD and a very limited number of trials that have examined its safety and efficacy for this indication. Although both clinical trials and real-world observational studies have strengths and limitations, randomised controlled trials continue to be considered the gold standard in medical research (although see^42^). However, given widespread prescription of medicinal cannabis and an apparent reluctance of funding agencies and sponsors to fund rigorous trials of its safety and efficacy, there should be greater recognition of the advantages of real-world data for advancing knowledge in this area.

## Data Availability

The data that support the findings of this study are available from the corresponding author (M.T.L.) on reasonable request. The data are not publicly available owing to the sensitive nature of the data and concerns that widespread release could compromise the privacy of research participants.

## References

[1] Lynskey MT, Schlag AK, Athanasiou-Fragkouli A, Badcock D, Nutt DJ. Characteristics of and 3-month health outcomes for people seeking treatment with prescribed cannabis: real-world evidence from project Twenty21. Drug Sci Policy Law 2023; 9: 20503245231167373.

[2] Schlag AK, Lynskey M, Fayaz A, Athanasiou-Fragkouli A, Brandner B, Haja B, et al. Characteristics of people seeking prescribed cannabinoids for the treatment of chronic pain: evidence from Project Twenty 21. Front Pain Res Lausanne 2022; 3: 891498. 35775024 10.3389/fpain.2022.891498 PMC9237624

[3] Ashare RL, Kelly E, Hajjar ER, Pant S, Meghani SH, Worster B. Characterizing anxiety, pain, sleep, and quality of life among patients in a state medical marijuana program. Complement Ther Clin Pract 2022; 48: 101612. 35667225 10.1016/j.ctcp.2022.101612

[4] Leung J, Chan G, Stjepanović D, Chung JYC, Hall W, Hammond D. Prevalence and self-reported reasons of cannabis use for medical purposes in USA and Canada. Psychopharmacology (Berl) 2022; 239: 1509–19. 35020045 10.1007/s00213-021-06047-8 PMC9110511

[5] Mahabir VK, Merchant JJ, Smith C, Garibaldi A. Medical cannabis use in the United States: a retrospective database study. J Cannabis Res 2020; 2(1): 32. 33526110 10.1186/s42238-020-00038-w PMC7819290

[6] Sznitman SR. Trends in medical cannabis licensure, Israel, 2013–2018. Drug Alcohol Rev 2020; 39: 763–7. 32557909 10.1111/dar.13116

[7] Forsythe ML, Boileau AJ. Use of cannabinoids for the treatment of patients with post-traumatic stress disorder. J Basic Clin Physiol Pharmacol 2021; 33: 121–32. 33662194 10.1515/jbcpp-2020-0279

[8] Lookfong NA, Raup-Konsavage WM, Silberman Y. Potential utility of cannabidiol in stress-related disorders. Cannabis Cannabinoid Res 2023; 8: 230–40. 36409719 10.1089/can.2022.0130 PMC10061337

[9] Bourke SL, Schlag AK, O'Sullivan SE, Nutt DJ, Finn DP. Cannabinoids and the endocannabinoid system in fibromyalgia: a review of preclinical and clinical research. Pharmacol Ther 2022; 240: 108216. 35609718 10.1016/j.pharmthera.2022.108216

[10] Suraev A, Mills L, Abelev SV, Arkell TR, Lintzeris N, McGregor IS. Medical cannabis use patterns for sleep disorders in Australia: results of the cross-sectional CAMS-20 survey. Nat Sci Sleep 2023; 15: 245–55. 37090897 10.2147/NSS.S390583 PMC10120832

[11] Blessing EM, Steenkamp MM, Manzanares J, Marmar CR. Cannabidiol as a potential treatment for anxiety disorders. Neurotherapeutics 2015; 12: 825–36. 26341731 10.1007/s13311-015-0387-1 PMC4604171

[12] Elmes MW, Kaczocha M, Berger WT, Leung K, Ralph BP, Wang L, et al. Fatty acid-binding proteins (FABPs) are intracellular carriers for Δ9-tetrahydrocannabinol (THC) and cannabidiol (CBD). J Biol Chem 2015; 290: 8711–21. 25666611 10.1074/jbc.M114.618447 PMC4423662

[13] Black N, Stockings E, Campbell G, Tran LT, Zagic D, Hall WD, et al. Cannabinoids for the treatment of mental disorders and symptoms of mental disorders: a systematic review and meta-analysis. Lancet Psychiatry 2019; 6: 995–1010. 31672337 10.1016/S2215-0366(19)30401-8 PMC6949116

[14] Jetly R, Heber A, Fraser G, Boisvert D. The efficacy of nabilone, a synthetic cannabinoid, in the treatment of PTSD-associated nightmares: a preliminary randomized, double-blind, placebo-controlled cross-over design study. Psychoneuroendocrinology 2015; 51: 585–8. 25467221 10.1016/j.psyneuen.2014.11.002

[15] Bonn-Miller MO, Sisley S, Riggs P, Yazar-Klosinski B, Wang JB, Loflin MJE, et al. The short-term impact of 3 smoked cannabis preparations versus placebo on PTSD symptoms: a randomized cross-over clinical trial. PLoS One 2021; 16(3): e0246990. 33730032 10.1371/journal.pone.0246990 PMC7968689

[16] Conybeare D, Behar E, Solomon A, Newman MG, Borkovec TD. The PTSD Checklist-Civilian Version: reliability, validity, and factor structure in a nonclinical sample. J Clin Psychol 2012; 68: 699–713. 22517497 10.1002/jclp.21845

[17] Pillai M, Erridge S, Bapir L, Nicholas M, Dalavaye N, Holvey C, et al. Assessment of clinical outcomes in patients with post-traumatic stress disorder: analysis from the UK medical Cannabis registry. Expert Rev Neurother 2022; 22: 1009–18. 36503404 10.1080/14737175.2022.2155139

[18] Creamer M, Bell R, Failla S. Psychometric properties of the impact of event scale - revised. Behav Res Ther 2003; 41: 1489–96. 14705607 10.1016/j.brat.2003.07.010

[19] Nacasch N, Avni C, Toren P. Medical cannabis for treatment-resistant combat PTSD. Front Psychiatry 2022; 13: 1014630. 36741572 10.3389/fpsyt.2022.1014630 PMC9893003

[20] Rothwell PM. Factors that can affect the external validity of randomised controlled trials. PLoS Clin Trials 2006; 1(1): e9. 16871331 10.1371/journal.pctr.0010009 PMC1488890

[21] Schlag AK, Zafar RR, Lynskey MT, Athanasiou-Fragkouli A, Phillips LD, Nutt DJ. The value of real world evidence: the case of medical cannabis. Front Psychiatry 2022; 13: 1027159. 36405915 10.3389/fpsyt.2022.1027159 PMC9669276

[22] Leeman RF, Hefner K, Frohe T, Murray A, Rosenheck RA, Watts BV, et al. Exclusion of participants based on substance use status: findings from randomized controlled trials of treatments for PTSD. Behav Res Ther 2017; 89: 33–40. 27846419 10.1016/j.brat.2016.10.006

[23] Zimmerman M, Balling C, Chelminski I, Dalrymple K. Have treatment studies of depression become even less generalizable? Applying the inclusion and exclusion criteria in placebo-controlled antidepressant efficacy trials published over 20 years to a clinical sample. Psychother Psychosom 2019; 88: 165–70. 31096246 10.1159/000499917

[24] Brady KT, Killeen TK, Brewerton T, Lucerini S. Comorbidity of psychiatric disorders and posttraumatic stress disorder. J Clin Psychiatry 2000; 61(suppl 7): 22–32. 10795606

[25] Sakal C, Lynskey M, Schlag AK, Nutt DJ. Developing a real-world evidence base for prescribed cannabis in the United Kingdom: preliminary findings from project Twenty21. Psychopharmacology (Berl) 2022; 239: 1147–55. 33970291 10.1007/s00213-021-05855-2

[26] Kroenke K, Spitzer RL, Williams JB. The PHQ-9: validity of a brief depression severity measure. J Gen Intern Med 2001; 16: 606–13. 11556941 10.1046/j.1525-1497.2001.016009606.x PMC1495268

[27] EuroQol Research Foundation. EQ-5D-5L User Guide. EuroQol, 2019.

[28] Buysse DJ, Reynolds CF, Monk TH, Berman SR, Kupfer DJ. The Pittsburgh Sleep Quality Index: a new instrument for psychiatric practice and research. Psychiatry Res 1989; 28: 193–213. 2748771 10.1016/0165-1781(89)90047-4

[29] IBM Corp. IBM SPSS Statistics for Windows, Version 29.0. IBM Corp, 2022.

[30] Arkell TR, Downey LA, Hayley AC, Roth S. Assessment of medical cannabis and health-related quality of life. JAMA Netw Open 2023; 6(5): e2312522. 37159196 10.1001/jamanetworkopen.2023.12522 PMC10170337

[31] Kline AC, Cooper AA, Rytwinski NK, Feeny NC. The effect of concurrent depression on PTSD outcomes in trauma-focused psychotherapy: a meta-analysis of randomized controlled trials. Behav Ther 2021; 52: 250–66. 33483121 10.1016/j.beth.2020.04.015 PMC7826446

[32] Mitchell JM, Bogenschutz M, Lilienstein A, Harrison C, Kleiman S, Parker-Guilbert K, et al. MDMA-assisted therapy for severe PTSD: a randomized, double-blind, placebo-controlled phase 3 study. Nat Med 2021; 27: 1025–33. 33972795 10.1038/s41591-021-01336-3 PMC8205851

[33] Aderka IM, Gillihan SJ, McLean CP, Foa EB. The relationship between posttraumatic and depressive symptoms during prolonged exposure with and without cognitive restructuring for the treatment of posttraumatic stress disorder. J Consult Clin Psychol 2013; 81: 375–82. 23339538 10.1037/a0031523

[34] Liverant GI, Suvak MK, Pineles SL, Resick PA. Changes in posttraumatic stress disorder and depressive symptoms during cognitive processing therapy: evidence for concurrent change. J Consult Clin Psychol 2012; 80: 957–67. 23067427 10.1037/a0030485

[35] Mangoo S, Erridge S, Holvey C, Coomber R, Barros DAR, Bhoskar U, et al. Assessment of clinical outcomes of medicinal cannabis therapy for depression: analysis from the UK Medical Cannabis Registry. Expert Rev Neurother 2022; 22: 995–1008. 36573268 10.1080/14737175.2022.2161894

[36] Flory JD, Yehuda R. Comorbidity between post-traumatic stress disorder and major depressive disorder: alternative explanations and treatment considerations. Dialogues Clin Neurosci 2015; 17: 141–50. 26246789 10.31887/DCNS.2015.17.2/jflory PMC4518698

[37] Stockings E, Campbell G, Hall WD, Nielsen S, Zagic D, Rahman R, et al. Cannabis and cannabinoids for the treatment of people with chronic noncancer pain conditions: a systematic review and meta-analysis of controlled and observational studies. Pain 2018; 159: 1932–54. 29847469 10.1097/j.pain.0000000000001293

[38] Simei JLQ, Souza JDR, Lisboa JR, Campos AC, Guimarães FS, Zuardi A, et al. Does the ‘entourage effect’ in cannabinoids exist? A narrative scoping review. Cannabis Cannabinoid Res [Epub ahead of print] 3 Aug 2023. Available from: 10.1089/can.2023.0052. 37535820

[39] Smith RT, Gruber SA. Contemplating cannabis? The complex relationship between cannabinoids and hepatic metabolism resulting in the potential for drug-drug interactions. Front Psychiatry 2023; 13: 1055481. 10.3389/fpsyt.2022.1055481 PMC9871609 36704740

[40] Doohan PT, Oldfield LD, Arnold JC, Anderson LL. Cannabinoid interactions with cytochrome P450 drug metabolism: a full-Spectrum characterization. AAPS J 2021; 23(4): 91. 34181150 10.1208/s12248-021-00616-7

[41] Zendulka O, Dovrtělová G, Nosková K, Turjap M, Šulcová A, Hanuš L, et al. Cannabinoids and cytochrome P450 interactions. Curr Drug Metab 2016; 17: 206–26. 26651971 10.2174/1389200217666151210142051

[42] Szigeti B, Phillips LD, Nutt D. Bayesian analysis of real-world data as evidence for drug approval: remembering Sir Michael Rawlins. Br J Clin Pharmacol 2023; 89: 2646–8. 37455605 10.1111/bcp.15841

